# Supplementary material for: Fatty Acid Composition and Oxidative Stability of the Lipid Fraction of Skin-On and Skinless Fillets of Prussian Carp (Carassius gibelio)
Source: Animals (Basel). 2020 Apr 30;10(5):778. doi: 10.3390/ani10050778 (PMC7278405; doi:10.3390/ani10050778)
Supplement: Supplementary file 1 [file animals-10-00778-s001.pdf]

Table S1. Fatty acid profile (% fatty acids) and content (mg·100 g^{-1} fillet) of Prussian Carp (*Carassius gibelio*) fillets (mean ± SD).

Individual Fatty Acids	% Fatty Acids		mg·100 g^{-1} fillet	
	Skin-On Fillet	Skinless Fillet	Skin-On Fillet	Skinless Fillet
C8:0	0.00^{a} ± 0.00	0.01^{b} ± 0.00	0.03 ± 0.04	0.06 ± 0.01
C10:0	0.01^{A} ± 0.00	0.02^{B} ± 0.00	0.23 ± 0.08	0.18 ± 0.05
C11:0	0.01 ± 0.00	0.01 ± 0.00	0.37^{y} ± 0.13	0.13^{x} ± 0.02
C12:0	0.12^{B} ± 0.00	0.11^{A} ± 0.00	4.11^{Y} ± 0.10	1.06^{X} ± 0.03
C13:0	0.03 ± 0.01	0.02 ± 0.00	1.13^{Y} ± 0.31	0.24^{X} ± 0.04
C14:0	2.19^{B} ± 0.05	1.97^{A} ± 0.02	76.73^{Y} ± 1.89	19.63^{X} ± 0.15
C15:0	0.43^{b} ± 0.01	0.41^{a} ± 0.00	14.97^{Y} ± 0.31	4.10^{X} ± 0.03
C16:0	14.00^{A} ± 0.24	14.72^{B} ± 0.22	490.55^{Y} ± 8.32	146.51^{X} ± 2.22
C17:0	0.54^{A} ± 0.01	0.58^{B} ± 0.00	18.91^{Y} ± 0.35	5.75^{X} ± 0.02
C18:0	3.40^{A} ± 0.04	4.14^{B} ± 0.05	119.06^{Y} ± 1.44	41.20^{X} ± 0.52
C20:0	0.17 ± 0.01	0.18 ± 0.01	6.07^{Y} ± 0.52	1.79^{X} ± 0.14
C21:0	0.02 ± 0.02	0.01 ± 0.01	0.85 ± 0.69	0.11 ± 0.10
C22:0	0.03 ± 0.01	0.04 ± 0.02	1.01^{Y} ± 0.22	0.39^{X} ± 0.22
C23:0	0.03 ± 0.03	0.03 ± 0.02	1.22 ± 0.94	0.32 ± 0.23
C24:0	0.39^{A} ± 0.02	0.56^{B} ± 0.01	13.63^{Y} ± 0.63	5.62^{X} ± 0.07
ΣSFA	21.37^{A} ± 0.29	22.82^{B} ± 0.15	748.87^{Y} ± 10.25	227.09^{X} ± 1.49
C14:0*iso*	0.07 ± 0.00	0.06 ± 0.01	2.30^{Y} ± 0.10	0.64^{X} ±0.07
C15:0*iso*	0.10^{B} ± 0.00	0.07^{A} ± 0.01	3.66^{Y} ± 0.10	0.74^{X} ±0.06
C15:0*ante*	0.16^{A} ± 0.00	0.19^{B} ± 0.01	5.67^{Y} ± 0.17	1.88^{X} ± 0.10
C16:0*iso*	0.37^{A} ± 0.01	0.41^{B} ± 0.01	13.09^{Y} ± 0.23	4.04^{X} ± 0.10
C17:0*iso*	1.61^{A} ± 0.03	2.31^{B} ± 0.06	56.52^{Y} ± 1.10	22.96^{X} ±0.60
C17:0*ante*	1.03^{A} ± 0.05	1.40^{B} ± 0.01	35.94^{Y} ± 1.59	13.98^{X} ± 0.09
C18:0*iso*	0.45^{B} ± 0.02	0.35^{A} ± 0.02	15.89^{Y} ± 0.64	3.50^{X} ± 0.20
ΣBCFA	3.80^{A} ± 0.02	4.79^{B} ± 0.06	133.07^{Y} ± 0.75	47.72^{X} ± 0.61
C14:1	0.38 ± 0.01	0.37 ± 0.01	13.26^{Y} ± 0.28	3.64^{X} ± 0.05
C15:1	0.24^{A} ± 0.01	0.43^{B} ± 0.01	8.50^{Y} ± 0.30	4.24^{X} ± 0.09
C16:1*cis*13	0.05 ± 0.01	0.05 ± 0.01	1.85^{Y} ± 0.29	0.51^{X} ± 0.10
C16:1n-7	13.35^{B} ± 0.21	10.43^{A} ± 0.19	467.96^{Y} ± 7.28	103.84^{X} ± 1.85
C16:1n-9	1.09 ± 0.02	1.04 ± 0.03	38.07^{Y} ± 0.71	10.35^{X} ± 0.34
C17:1*cis*9	0.53 ± 0.02	0.51 ± 0.00	18.66^{Y} ± 0.56	5.13^{X} ± 0.05
C17:1*isomer*	0.21^{A} ± 0.01	0.25^{B} ± 0.01	7.46^{Y} ± 0.32	2.44^{X} ± 0.11
C18:1*cis*11	6.66^{B} ± 0.05	6.22^{A} ± 0.02	233.50^{Y} ± 1.87	61.93^{X} ± 0.18
C18:1*cis*12	0.02 ± 0.02	0.03 ± 0.02	0.77 ± 0.66	0.30 ± 0.18
C18:1*cis*13	0.18 ± 0.01	0.19 ± 0.02	6.22^{Y} ± 0.44	1.92^{X} ± 0.24
C18:1n-9	13.98 ± 0.08	14.02 ± 0.05	489.85^{Y} ± 2.81	139.56^{X} ± 0.46
C20:1n-7	0.02 ± 0.01	0.02 ± 0.00	0.71^{y} ± 0.25	0.24^{x} ± 0.03
C20:1n-9	0.17 ± 0.02	0.17 ± 0.04	5.96^{Y} ± 0.54	1.67^{X} ± 0.37
C20:1n-11	0.79 ± 0.06	0.75 ± 0.01	27.59^{Y} ± 2.02	7.47^{X} ± 0.08
C22:1n-9	0.02^{a} ± 0.00	0.03^{b} ± 0.00	0.76^{Y} ± 0.10	0.27^{X} ± 0.03
C22:1n-11	0.05 ± 0.02	0.04 ± 0.01	1.68^{y} ± 0.73	0.37^{x} ± 0.06
C24:1n-9	0.05 ± 0.01	0.08 ± 0.02	1.82^{y} ± 0.51	0.84^{x} ± 0.25
ΣMUFA	38.48^{B} ± 0.16	35.31^{A} ± 0.18	1348.62^{Y} ± 5.70	351.47^{X} ± 1.83
C16:1*trans*9	0.35^{B} ± 0.01	0.28^{A} ± 0.02	12.14^{Y} ± 0.46	2.80^{X} ± 0.19
C18:1*trans*6/7	0.16^{a} ± 0.02	0.19^{b} ± 0.01	5.49^{Y} ± 0.64	1.92^{X} ± 0.10
C18:1*trans*9	0.05 ± 0.01	0.05 ± 0.01	1.77^{Y} ± 0.20	0.49^{X} ± 0.09

C18:1*trans*10	0.05 ± 0.01	0.07 ± 0.01	1.83^{Y} ± 0.40	0.69^{X} ± 0.13
C18:1*trans*16	0.08 ± 0.01	0.09 ± 0.01	2.76^{Y} ± 0.25	0.87^{X} ± 0.13
ΣC18:2*trans*	0.38 ± 0.04	0.38 ± 0.03	13.16^{Y} ± 1.52	3.81^{X} ± 0.28
ΣTFA	1.06 ± 0.06	1.06 ± 0.06	37.15^{Y} ± 2.17	10.58^{X} ± 0.59
C18:2n-6	2.46 ± 0.02	2.48 ± 0.01	86.23^{Y} ± 0.58	24.66^{X} ± 0.06
C18:3n-6	0.28^{B} ± 0.01	0.19^{A} ± 0.01	9.89^{Y} ± 0.38	1.86^{X} ± 0.12
C20:2n-6	0.38^{A} ± 0.02	0.49^{B} ± 0.01	13.29^{Y} ± 0.73	4.85^{X} ± 0.14
C20:3n-6	0.32^{A} ± 0.02	0.41^{B} ± 0.01	11.30^{Y} ± 0.67	4.10^{X} ± 0.14
C20:4n-6	2.85^{A} ± 0.04	3.89^{B} ± 0.05	99.91^{Y} ± 1.39	38.73^{X} ± 0.53
C22:4n-6	0.29^{B} ± 0.01	0.24^{A} ± 0.02	10.18^{Y} ± 0.51	2.39^{X} ± 0.20
C22:5n-6	0.18^{A} ± 0.02	0.26^{B} ± 0.03	6.36^{Y} ± 0.81	2.59^{X} ± 0.30
Σn-6	6.77^{A} ± 0.06	7.96^{B} ± 0.06	237.16^{Y} ± 2.23	79.20^{X} ± 0.58
C18:3n-3	3.40^{B} ± 0.03	2.62^{A} ± 0.01	119.22 ^{Y} ± 0.91	26.08^{X} ± 0.11
C18:4n-3	0.47^{B} ± 0.01	0.25^{A} ± 0.00	16.31^{Y} ± 0.36	2.49^{X} ± 0.04
C20:3n-3	0.50^{B} ± 0.01	0.44^{A} ± 0.01	17.53^{Y} ± 0.36	4.38^{X} ± 0.14
C20:4n-3	0.66 ± 0.06	0.62 ± 0.03	23.30^{Y} ± 2.08	6.18^{X} ± 0.27
C20:5n-3 (EPA)	11.80^{B} ± 0.17	10.58^{A} ± 0.18	413.69^{Y} ± 5.80	105.28^{X} ± 1.84
C22:5n-3	3.07^{a} ± 0.10	3.25^{b} ± 0.06	107.66^{Y} ± 3.41	32.31^{X} ± 0.56
C22:6n-3 (DHA)	6.12^{A} ± 0.19	8.64^{B} ± 0.13	214.35^{Y} ± 6.66	86.04^{X} ± 1.31
Σn-3	26.02 ± 0.47	26.40 ± 0.42	912.07^{Y} ± 16.50	262.75^{X} ± 4.17
ΣC18:3*isomers*	0.87^{B} ± 0.04	0.73^{A} ± 0.02	30.55^{Y} ± 1.57	7.23^{X} ± 0.21
C16:2n-2	1.15^{B} ± 0.01	0.75^{A} ± 0.01	40.14^{Y} ± 0.51	7.47^{X} ± 0.10
C16:2n-4	0.07^{B} ± 0.01	0.05^{A} ± 0.01	2.40^{Y} ± 0.24	0.46^{X} ± 0.09
C16:3n-4	0.99^{B} ± 0.03	0.69^{A} ± 0.02	34.68^{Y} ± 0.96	6.87^{X} ± 0.25
C16:4n-1	0.04 ± 0.01	0.04 ± 0.01	1.35^{y} ± 0.48	0.36^{x} ± 0.05
C20:2n-9	0.07 ± 0.02	0.07 ± 0.03	2.55^{Y} ± 0.58	0.71^{X} ± 0.26
C22:2*cis*	0.01 ± 0.01	0.01 ± 0.01	0.42 ± 0.29	0.14 ± 0.08
ΣUFA	74.84^{B} ± 0.31	72.39^{A} ± 0.19	2623.09^{Y} ± 10.76	720.45^{X} ± 1.87
ΣPUFA	36.36 ± 0.46	37.07 ± 0.37	1274.47^{Y} ± 16.26	368.98^{X} ± 3.65
Σn-3/Σn-6	3.85^{B} ± 0.05	3.32^{A} ± 0.03	–	–
ΣPUFA/ΣSFA	1.70^{b} ± 0.05	1.62^{a} ± 0.03	–	–
EPA/DHA	1.93^{B} ± 0.05	1.22^{A} ± 0.00	–	–

Mean values in rows with different letters differ statistically significantly: a, b: $p \leq 0.05$, A, B: $p \leq 0.01$.
Mean values in rows with different letters differ statistically significantly: x, y: $p \leq 0.05$, X, Y: $p \leq 0.01$.
SFA: total saturated fatty acids; BCFA: total branched-chain fatty acids; MUFA: total monounsaturated fatty acids; TFA: total trans fatty acids; UFA: total unsaturated fatty acids; PUFA: total polyunsaturated fatty acids; Σn-6: total n-6 fatty acids; Σn-3: total n-3 fatty acids; EPA: eicosapentaenoic acid; DHA: docosahesaenoic acid
